# Repeated exposure to trauma narratives and professional quality of life in palliative and end-of-life healthcare providers

**DOI:** 10.1017/S1478951526101783

**Published:** 2026-03-05

**Authors:** Suzanne A Brier, Amy L Nadel, Charlotte Stone, Rebecca M Schwartz

**Affiliations:** 1 Northwell Health, New York, NY, USA; 2 Department of Psychiatry, Donald and Barbara Zucker School of Medicine at Hofstra/Northwell, New York, NY, USA; 3 Division of Pediatric Hematology/Oncology and Stem Cell Transplantation, Cohen Children’s Medical Center, Northwell Health, New York, NY, USA; 4 Division of Geriatrics and Palliative Medicine, Lenox Hill Hospital, Northwell Health, New York, NY, USA; 5 Office of Medical Education and Professionalism (OMEP), Zucker School of Medicine at Hofstra/Northwell, Northwell Health, New York, NY, USA; 6 Feinstein Institutes for Medical Research, Northwell Health, Manhasset, New York, NY, USA

**Keywords:** Vicarious trauma, secondary traumatic stress, compassion satisfaction, resilience, organizational support, end-of-life care

## Abstract

**Objectives:**

This study examined how repeated exposure to trauma narratives influences professional quality of life, including burnout, secondary traumatic stress (STS), and compassion satisfaction (CS), among end-of-life healthcare providers. The moderating roles of resilience and both organizational and personal support were also tested.

**Methods:**

A cross-sectional online survey was completed by 507 healthcare providers working in hospice, oncology, or other end-of-life settings. Participants completed validated self-report measures assessing exposure to trauma narratives (Vicarious Trauma Scale [VTS]), professional quality of life (ProQOL-5), resilience (STARS-6), organizational support (SPOS), and social support (MSPSS). Hierarchical regression and moderation analyses were conducted to evaluate main and interaction effects.

**Results:**

Greater exposure to trauma narratives was significantly associated with higher burnout (*β* = .37, *p* < .001) and STS (*β* = .42, *p* < .001), and with lower CS (*β* = −.13, *p* = .004). Higher resilience and organizational support predicted greater CS and lower burnout and STS. A significant VTS × resilience interaction indicated that resilience buffered the association between exposure to trauma narratives and STS (*β* = −.10, *p* = .009).

**Conclusions:**

Repeated exposure to trauma narratives is a meaningful occupational stressor for end-of-life clinicians. Resilience and organizational support appear to protect against the negative impact of trauma exposure and promote CS, highlighting key multilevel targets for trauma-informed workforce interventions.

**Significance of results:**

This study addresses a critical gap by clarifying how repeated trauma narratives specifically influence burnout, Secondary Traumatic Stress (STS), and Compassion Satisfaction (CS) within the unique context of end-of-life care. The results provide a nuanced framework for understanding how clinicians maintain empathic presence despite chronic emotional demands. Furthermore, by identifying specific resilience factors and support systems that buffer against psychological distress, these findings offer actionable insights for developing targeted interventions to mitigate long-term professional harm.

Healthcare providers working in oncology, hospice, and other end-of-life care contexts are routinely exposed to suffering, loss, and grief. These environments require clinicians to engage in complex medical care, sustained emotional labor, the delivery of devastating news, and support for families, all while witnessing prolonged or painful deaths (Pereira et al. 2011; Granek et al. 2012; Sinclair et al. 2017; Cañadas-de la Fuente et al. 2018). While these encounters are often normalized within clinical settings, they often feel traumatic or psychologically destabilizing (Sinclair et al. 2017; Cañadas-de la Fuente et al. 2018). The cumulative exposure to such trauma narratives, particularly when compounded by a lack of adequate institutional or interpersonal support, has the potential to erode providers’ emotional well-being and contribute to chronic distress (Delaney 2018; West et al. 2018).

Prior research has identified elevated levels of burnout and secondary traumatic stress (STS) among healthcare professionals working in emotionally demanding settings (Duarte and Pinto-Gouveia 2017; Orrù et al. 2021). The professional quality of life (ProQOL) model remains one of the most widely used frameworks for conceptualizing these outcomes. It delineates 3 primary domains: burnout, STS, and compassion satisfaction (CS). CS captures the positive emotional rewards and sense of meaning that providers derive from helping others (Stamm 2010). Although this framework is well-established, the specific mechanisms by which repeated trauma exposure alters emotional functioning in relation to CS, emotional regulation, and resilience remain underexplored, particularly among end-of-life care clinicians. While foundational theories of secondary traumatization (Figley 1995; Bride et al. 2004) have found that indirect exposure to trauma can result in posttraumatic symptoms, more recent work has begun to consider how these experiences interact with cognitive-affective processes and systemic workplace factors (Alkema et al. 2008; Boyle 2018).

CS is a complex construct in end-of-life care. On the one hand, CS enables clinicians to build therapeutic rapport, respond empathetically, and navigate the emotional demands of caregiving. On the other hand, chronic emotional engagement in the face of unrelenting trauma exposure may become unsustainable, leading to emotional exhaustion, overidentification, or psychological detachment (Sinclair et al. 2017). Some clinicians experiencing compassion fatigue report symptoms such as heightened anxiety, hypervigilance, and concerns about their own health or the well-being of loved ones. Others describe a gradual emotional numbing or withdrawal that may allow short-term functioning but ultimately compromises psychological health. In more severe cases, clinicians may internalize the pain of their patients without adequate tools for regulation, leading compassion to become a source of suffering (Figley 1995; Duarte and Pinto-Gouveia 2017; Sinclair et al. 2017). Duarte and Pinto-Gouveia (2017) found that an imbalance between compassion and emotion regulation was associated with both STS and burnout. These findings underscore the importance of understanding CS as both a protective and risk factor.

Interpersonal and institutional support may play a moderating role in psychological outcomes among healthcare workers. Perceived support from supervisors, coworkers, and broader social networks has been shown to buffer the impact of repeated trauma exposure, particularly in clinical and caregiving environments (Killian 2008). For example, the protective role of perceived support in high-stress settings. Similarly, Killian (2008) focused on social workers engaged in trauma-intensive care, identifying supervisory, and peer validation as critical buffers. When clinicians feel validated, seen, and understood within their work environments, they are more likely to report psychological resilience and lower rates of emotional exhaustion. Conversely, isolation, invalidation, and inadequate systemic support may intensify emotional burdens and reduce providers’ capacity to remain present and empathically engaged. Individual differences in emotion regulation and resilience may further shape these trajectories (West et al. 2018). Although secondary traumatization and burnout have been documented among healthcare workers broadly, little is known about how repeated trauma narratives shape outcomes in oncology and hospice clinicians. The present study aims to fill this gap by examining the impact of repeated exposure on burnout, STS, and CS in end-of-life care, and by further exploring how resilience and support systems may buffer these effects. Findings will contribute to a more nuanced understanding of how end-of-life clinicians sustain an empathic presence in the face of chronic emotional demands, and which resources are most protective in mitigating long-term harm.

## Methods

### Study design

This study employed a cross-sectional, self-report survey design to examine the emotional impact of repeated trauma exposure among healthcare professionals working in high-intensity clinical settings, specifically oncology and hospice care. Trauma exposure was operationalized as the frequency and intensity of exposure to patient trauma narratives and end-of-life events.

The primary aim was to assess how trauma exposure relates to ProQOL outcomes, including burnout, STS, and CS. Additionally, the study examined whether perceived organizational support, social support, resilience, and emotion regulation moderate the effects of trauma exposure on these outcomes.

### Participants

The sample consisted of 507 physicians and nurses employed in end-of-life care (e.g., oncology, hospice, critical care, and intensive care units) across a large health system in the Northeast United States. Recruitment was facilitated through department listservs, physician well-being representatives, and direct outreach from unit leadership. A total of 2,374 contacts were sent information regarding the study with a survey link. Inclusion criteria required that participants provide direct care to patients at the end-of-life; all responses were anonymous and voluntary. The survey took approximately 5 minutes to complete and was administered through an email link to a REDCap survey and consent form. All participants completed electronic informed consent prior to participation. The study was approved by the Institutional Review Board. REDCap is a HIPAA-compliant, secure web platform. Participants could choose to enter their email addresses (not linked to their survey responses) into a raffle to win one of a $100 Amazon gift cards upon completion. One participant was selected as the winner.

## Measures

### Independent variable

**Vicarious trauma symptoms.** Vicarious trauma was assessed using the Vicarious Trauma Scale (VTS; Vrklevski and Franklin 2008). The VTS is a 10-item self-report instrument that captures the extent to which indirect exposure to others’ traumatic experiences affects clinicians’ emotional and cognitive functioning. Items are rated on a 7-point Likert scale from 1 (strongly disagree) to 7 (strongly agree), with higher scores indicating greater vicarious trauma symptoms. The VTS has demonstrated strong internal consistency reliability in prior studies (Cronbach’s *α* = .88) and has been validated with a range of healthcare providers. The VTS has demonstrated strong internal consistency reliability in prior studies (Cronbach’s *α* = .88) and has been validated in samples of healthcare providers and other helping professionals (Jenkins and Baird 2002; Kadambi and Truscott 2004; Vrklevski and Franklin 2008).

## Dependent variables

### CS, burnout, and STS

These outcomes were measured using the Professional Quality of Life Scale, Version 5 (ProQOL-5; Stamm 2010). The ProQOL-5 includes 30 items divided into three 10-item subscales: CS, burnout, and STS. Participants respond using a 5-point Likert scale ranging from 1 (never) to 5 (very often). Each subscale yields a continuous score, with higher scores on CS reflecting greater fulfillment in one’s professional role, and higher scores on burnout or STS indicating higher emotional exhaustion or trauma-related distress, respectively. Internal consistencies for the ProQOL-5 subscales in healthcare samples range from *α* = .88 for STS, *α* = .72 for burnout, and *α* = .88 for CS (Stamm 2010).

## Moderators

### Perceived social support

The Multidimensional Scale of Perceived Social Support (MSPSS; Zimet et al. 1988) was used to measure subjective social support across 3 domains: family, friends, and significant others. It contains 12 items rated on a 7-point Likert scale from 1 (very strongly disagree) to 7 (very strongly agree). Higher scores indicate greater perceived support. The MSPSS has consistently demonstrated high internal consistency reliability (*α* = .88–.92) and good construct validity across clinical and non-clinical populations (Zimet et al. 1988; Canty-Mitchell and Zimet 2000).

### Perceived organizational support

Organizational support was assessed using the Survey of Perceived Organizational Support (SPOS-4; Eisenberger et al. 1986). This abbreviated 4-item version measures the extent to which employees feel that their organization values their contributions and cares about their well-being. Each item is rated on a 7-point Likert scale from 1 (strongly disagree) to 7 (strongly agree). Higher total scores reflect greater perceived organizational support. Internal consistency for the SPOS-4 in prior healthcare samples has ranged from *α* = .84 to *α* = .90 (Eisenberger et al. 1986; Rhoades and Eisenberger 2002).

### Resilience

Resilience was measured using the State Assessment of Resilience Scale (STARS-6) (Lock et al. 2020). STARS-6 is a 6-item self-report measure designed to assess current, situational resilience (state-level). Respondents indicate agreement on a 5-point Likert scale ranging from 1 (strongly disagree) to 5 (strongly agree). Higher scores represent greater psychological resilience at the time of assessment. The STARS-6 has demonstrated acceptable psychometric properties (*α* = .76–.81) and strong factorial validity in healthcare settings (Lock et al. 2020).

### Data analysis

Descriptive statistics were computed for all study variables. Means and standard deviations were reported for continuous variables (e.g., age, years of experience, VTS, MSPSS, SPOS, STARS, and ProQOL subscales), and frequencies and percentages were reported for categorical variables (e.g., gender, race/ethnicity, education, role, work setting, employment status).

Bivariate analyses included Pearson’s correlations between continuous predictors and the 3 outcomes: CS, burnout, and STS. Group differences across demographic variables were examined using independent-samples *t*-tests (2 categories) or 1-way ANOVAs (3 or more categories). For clarity of presentation, continuous predictors were also dichotomized at the median in Table 1, although all inferential analyses retained these variables in their continuous form.

**Table 1.** Means (standard deviations) of compassion satisfaction, burnout, and secondary traumatic stress by predictor variables

Variable	CS *M* (*SD*)	Burnout *M* (*SD*)	STS *M* (*SD*)
**Age, mean (*SD*)**	42.27 (13.27)*	42.46 (13.28)*	42.52 (13.29)
**Years of experience, mean (*SD*)**	15.62 (11.27)*	15.75 (11.27)*	15.80 (11.28)
**Gender**			
Female (ref)	38.86 (5.29)	30.32 (3.10)	22.76 (6.40)
Male	38.67 (5.15)	29.67 (2.54)	20.86 (5.29)
Prefer not to answer	40.75 (5.27)	32.00 (2.05)	22.25 (2.21)
**Race**			
White (ref)	38.89 (5.14)	30.33 (3.06)	22.49 (6.46)
Black	38.31 (6.71)	27.59 (2.69)*	20.58 (5.93)
Asian	38.62 (5.32)	30.30 (2.65)	23.26 (5.27)
Multiracial	30.00 (9.64)	29.67 (1.15)	21.33 (3.78)
Other	40.27 (4.57)	30.65 (3.10)	22.08 (4.18)
Prefer not to say	38.86 (5.65)	31.62 (3.66)	23.29 (4.18)
**Hispanic ethnicity**			
Yes	40.45 (4.58)	30.23 (3.59)	21.23 (8.29)
No/Prefer not to say (ref)	38.80 (5.29)	30.27 (3.04)	22.58 (6.22)
**Education**			
Associate’s degree	38.53 (6.53)	28.93 (2.40)	21.33 (6.92)
Bachelor’s degree (ref)	38.24 (5.16)	30.44 (3.07)	23.73 (6.60)
Master’s degree	39.34 (5.16)	30.53 (3.00)	22.21 (5.82)
Doctoral degree	39.89 (5.35)	29.63 (3.03)	20.21 (5.29)
**Professional role**			
Nurse (ref)	38.43 (5.33)	30.47 (3.02)	23.42 (6.51)
Physician	39.70 (5.02)	29.86 (3.25)	20.25 (5.33)
Nurse practitioner	39.61 (4.47)	29.59 (1.74)	19.83 (4.69)
Physician assistant	39.35 (5.66)	31.04 (3.60)	24.00 (5.87)
Social worker	37.25 (4.43)	28.71 (3.50)	19.38 (4.34)
Other HCW	37.88 (6.15)	28.38 (2.97)	21.38 (5.76)
**Work setting**			
Oncology (ref)	39.46 (5.00)	30.61 (3.20)	22.78 (5.50)
Hospice	40.08 (4.99)	29.67 (3.08)	21.10 (7.03)
ICU	37.10 (5.25)	30.40 (2.87)	23.61 (7.04)
Critical care	38.20 (5.58)	30.68 (3.59)	22.80 (5.32)
Palliative care	41.03 (4.05)	29.48 (2.08)	20.62 (6.09)
Other	39.53 (5.87)	29.48 (3.03)	21.07 (5.90)
**Organizational support**			
Low (ref)	37.22 (5.75)***	31.12 (3.13)***	24.24 (6.78)***
High	39.93 (4.64)***	29.70 (2.87)***	21.42 (5.67)***
**Personal support**			
Low (ref)	36.63 (5.53)***	30.06 (3.12)	23.71 (6.94)***
High	39.95 (4.79)***	30.36 (3.02)	21.96 (5.86)***
**Resilience**			
Low (ref)	36.53 (5.42)***	31.16 (3.02)***	26.44 (6.89)***
High	40.11 (4.75)***	29.79 (2.96)***	20.45 (4.77)***
**Vicarious trauma**			
Low (ref)	39.98 (4.67)***	28.98 (2.58)***	19.18 (4.48)***
High	37.93 (5.57)***	31.33 (3.00)***	25.32 (6.21)***

*Note.* Values are presented as M (SD). “(ref)” denotes the reference category used in regression analyses. For categorical and median-split predictors, asterisks indicate significant between-group differences based on *t*-tests or 1-way ANOVAs. For continuous predictors (age, years of experience), asterisks indicate significance of Pearson’s correlation coefficients with the outcome variables.

* *p* < .05, ***p < .001.

Multiple linear regression analyses were then conducted to examine the associations between vicarious trauma symptoms (VTS) and each outcome (CS, burnout, STS), adjusting for relevant demographic covariates. Interaction terms were added to test whether resilience (STARS), perceived organizational support (SPOS), or perceived social support (MSPSS) moderated the association between VTS and outcomes, with a particular focus on STS. All analyses were conducted in IBM SPSS Statistics (Version 29.0.1), with significance set at *p* < .05.

## Results

### Descriptive statistics (univariate analyses)

Descriptive statistics for the 3 ProQOL subscales are presented in Table 1. CS scores were moderate on average (*M* = 38.5, *SD* = 6.2; range = 16–50), burnout scores were lower overall (*M* = 21.7, *SD* = 6.0; range = 10–50), and STS scores were lower than CS scores (*M* = 22.4, *SD* = 6.4; range = 10–50).

### Bivariate analyses

Table 1 presents group means, standard deviations, and bivariate associations between each of the outcomes and covariates.

Age was significantly negatively correlated with burnout, *r* = −.28, *p* < .001, and STS, *r* = −.40, *p* < .001, and weakly positively correlated with CS, *r* = .09, *p* = .07. Years of experience showed a similar pattern, with fewer years associated with higher burnout (*r* = −.20, *p* < .001) and higher STS (*r* = −.27, *p* < .001). Gender, education, professional role, work setting, and employment status were not significantly associated with any of the outcome variables in bivariate analyses. Race was generally not associated with outcome variables, with the exception that Black participants reported significantly lower burnout scores compared to White participants (*p* < .05).

For the main predictors, higher VTS scores were significantly associated with lower CS (*r* = −.36, *p* < .001) and higher burnout (*r* = .45, *p* < .001) and STS (*r* = .63, *p* < .001). Resilience was positively correlated with CS (*r* = .44, *p* < .001) and negatively correlated with burnout (*r* = −.29, *p* < .001) and STS (*r* = −.41, *p* < .001). Organizational support was also positively associated with CS (*r* = .46, *p* < .001) and negatively associated with burnout (*r* = −.35, *p* < .001) and STS (*r* = −.27, *p* < .001). Personal support was positively correlated with CS (*r* = .33, *p* < .001) and negatively correlated with burnout (*r* = −.14, *p* = .003) and STS (*r* = −.09, *p* = .06), though the latter association did not reach statistical significance.

Based on these results, age and years of experience were retained as covariates in the multivariable models. All 4 main predictors (VTS, resilience, organizational support, and personal support) were included in the multivariable analyses given their significant or theoretically relevant associations with the outcomes. Although personal support was not significantly associated with burnout in bivariate analyses, it was retained across models because of its theoretical importance in understanding support processes in trauma-exposed clinicians.

### Multivariable regression analyses

Separate multiple regression analyses were conducted to examine whether vicarious trauma symptoms (VTS), resilience, organizational support, and personal support were associated with CS, burnout, and STS, respectively, and whether these protective factors moderated the association between VTS and each outcome. The results of multiple regression analyses are presented in Table 2.

**Table 2.** Multiple regression analyses predicting compassion satisfaction, burnout, and secondary traumatic stress

Predictor	*β*	95% CI	*P*	*β*	95% CI	*p*	*β*	95% CI	*p*
**Model 1: Demographic covariates**									
Age	0.09	[−0.01, 0.19]	.07	−0.28***	[−0.42, −0.14]	<.001	−0.40***	[−0.50, −0.29]	<.001
Years of experience	0.06	[−0.04, 0.15]	.25	−0.11	[−0.24, 0.02]	.10	−0.13*	[−0.23, −0.02]	.02
Education	0.04	[−0.05, 0.13]	.38	−0.05	[−0.18, 0.08]	.44	−0.15**	[−0.25, −0.05]	.002
**Model 2: Model 1 + main predictors**									
Vicarious trauma symptoms	−0.13**	[−0.21, −0.04]	.004	0.37***	[0.28, 0.47]	<.001	0.42***	[0.34, 0.50]	<.001
Resilience	0.29***	[0.20, 0.38]	<.001	−0.12*	[−0.22, −0.02]	.02	−0.35***	[−0.43, −0.26]	<.001
Organizational support	0.34***	[0.25, 0.42]	<.001	−0.16***	[−0.26, −0.07]	<.001	−0.06	[−0.14, 0.02]	.14
Personal support	0.19***	[0.10, 0.28]	<.001	0.13**	[0.04, 0.22]	.005	−0.02	[−0.10, 0.06]	.59
**Model 3: Model 2 + interaction terms**									
VTS × resilience	−0.01	[−0.08, 0.06]	.69	−0.04	[−0.12, 0.04]	.30	−0.10**	[−0.17, −0.03]	.009
VTS × organizational support	0.02	[−0.05, 0.09]	.58	−0.01	[−0.09, 0.07]	.82	−0.07	[−0.14, 0.00]	.057
VTS × personal support	0.03	[−0.04, 0.10]	.42	0.02	[−0.06, 0.10]	.64	0.08*	[0.00, 0.15]	.045

*Note.* CS = compassion satisfaction; STS = secondary traumatic stress; VTS = vicarious trauma symptoms. Standardized beta coefficients (*β*) are reported. Brackets indicate 95% confidence intervals.

− *p* < .10, *p < .05, **p < .01, ***p < .001.

### Compassion satisfaction

The initial model including only demographic covariates (Model 1) accounted for a significant portion of variance in CS, *F*(8, 431) = 7.37, *p* < .001, *R*^2^ = .120 (12.0%). Adding VTS, resilience, organizational support, and personal support (Model 2) significantly improved the model, Δ*R*^2^ = .261, *F* change (4, 427) = 41.39, *p* < .001, for a total of 38.1% variance explained. In this model, higher resilience (*β* = .287, *p* < .001), greater organizational support (*β* = .338, *p* < .001), and greater personal support (*β* = .189, *p* < .001) each was associated with higher CS, while VTS was a significant negative predictor (*β* = −.127, *p* = .004). Adding the interaction terms (Model 3) did not explain additional variance, Δ*R*^2^ = .001, *F* change (3, 424) = 0.52, *p* = .692, and none of the interactions were significant.

### Burnout

The initial model containing only demographic variables (Model 1) accounted for 4.8% of the variance, *F*(8, 437) = 2.73, *p* = .006. Younger age was significantly negatively associated with burnout (*β* = −.279, *p* = .001). Adding the independent variables of interest (Model 2) significantly improved the model to 25.7% of variance explained, *F* change (4, 433) = 30.53, *p* < .001. VTS was the strongest positive predictor of burnout (*β* = .372, *p* < .001), while resilience (*β* = −.120, *p* = .020) and organizational support (*β* = −.163, *p* < .001) were associated with lower burnout. Personal support (*β* = .129, *p* = .005) was associated with higher burnout. The addition of interaction terms (Model 3) did not significantly improve the model, Δ*R*^2^ = .003, *F* change (3, 430) = 0.54, *p* = .658, and no interaction effects were significant.

### Secondary traumatic stress

Demographic covariates (Model 1) accounted for 10.9% of the variance, *F*(8, 439) = 6.74, *p* < .001 in STS. Younger age (*β* = −.398, *p* < .001), lower education (*β* = −.146, *p* = .002), and fewer years of experience (*β* = .185, *p* = .021) were associated with higher STS. Adding VTS and the putative protective factors (Model 2) explained 51.3% of the variance, Δ*R*^2^ = .403, *F* change (4, 435) = 90.08, *p* < .001. VTS was a strong positive predictor (*β* = .424, *p* < .001), and resilience was a strong negative predictor (*β* = −.353, *p* < .001). Adding the interaction terms explained an additional 1.4% of variance, *F* change (3, 432) = 4.36, *p* = .005. Two interactions were significant: the VTS × Resilience interaction (*β* = −.102, *p* = .009) indicated that resilience buffered the effect of trauma exposure on STS, and the VTS × Personal Support interaction (*β* = .075, *p* = .045) suggested that personal support unexpectedly intensified the effect of trauma exposure. The VTS × Organizational Support interaction was marginally significant (*β* = −.068, *p* = .057).

### Summary of findings

Bivariate analyses indicated that younger age and fewer years of experience were associated with higher burnout and STS, with a weak positive trend for CS. All 4 main predictors, VTS, resilience, organizational support, and personal support, were significantly correlated with at least 1 outcome, except for the association between personal support and STS, which was nonsignificant at the bivariate level.

Across all 3 multivariable models, vicarious trauma symptoms were consistently associated with poorer well-being, reflected by lower CS, higher burnout, and higher STS. Resilience and organizational support were generally protective across models, whereas personal support was beneficial for CS but associated with higher burnout and, in interaction with VTS, with higher STS. Significant moderation effects emerged only for STS, where resilience buffered and personal support intensified the impact of trauma exposure.

## Discussion

This study examined the relationships between vicarious trauma symptoms, protective factors, and ProQOL outcomes in healthcare providers working in oncology, hospice, and other end-of-life care settings. Across outcomes, vicarious trauma symptoms were consistently associated with lower CS, higher burnout, and higher STS, aligning with theoretical models of secondary traumatization (Figley 1995; Bride et al. 2004) and prior findings in trauma-heavy healthcare contexts (Duarte and Pinto-Gouveia 2017). These results underscore the cumulative emotional toll of repeated exposure to patient suffering and death, even for clinicians who perceive such encounters as “part of the job.”

In many end-of-life settings, the normalization of trauma exposure can obscure its gradual accumulation and long-term effects. The current findings add to a growing body of evidence suggesting that the psychological wear of repeated exposure does not necessarily plateau with experience and may manifest even among providers who remain deeply engaged and committed to their work (Sinclair et al. 2017; Boyle 2018). This highlights the need for proactive, preventive strategies rather than reactive interventions implemented only when distress becomes acute.

### The dual role of protective factors

Resilience and perceived organizational support were associated with higher CS and lower burnout, and resilience was also inversely associated with STS. Moreover, resilience moderated the association between vicarious trauma and STS, suggesting a buffering effect under conditions of high exposure. This supports prior research demonstrating that resilient providers are more likely to handle challenges adaptively and employ coping strategies that limit trauma-related distress (West et al. 2018). Importantly, this moderation effect indicates that resilience does more than simply show an association with well-being; it actively shapes how exposure translates into psychological outcomes.

Perceived organizational support also was associated with higher CS and lower burnout, consistent with the literature on the positive effects of feeling valued and supported by one’s institution (Eisenberger et al. 1986; Killian 2008). These findings reinforce the idea that workplace climate and leadership responsiveness are central determinants of whether emotional engagement in end-of-life care remains sustainable. However, organizational support did not significantly moderate the trauma–STS link, suggesting that even strong institutional climates may not fully buffer the more trauma-specific sequelae of repeated exposure. This distinction is critical for intervention design, pointing to the need for trauma-informed organizational practices beyond general support initiatives.

### The paradox of personal support

One of the more unexpected findings in this study was that personal support, while linked to greater CS, also was associated with higher burnout and, in combination with high vicarious trauma, intensified STS. At first glance, this seems contradictory. However, it becomes more understandable when viewed through frameworks that distinguish between perceived and received support.

Perceived support, the steady knowledge that help is there if needed, has consistently been shown to buffer stress across contexts. Received support, meaning the actual supportive acts, is more complex. It can be deeply helpful, but when it is highly visible and mismatched to the recipient’s needs, it can backfire (Maisel and Gable 2009). Visible support that feels misaligned can inadvertently heighten distress, trigger feelings of indebtedness, or make a person feel “on display” in their struggle. In contrast, invisible support, or help given subtly without drawing attention to it, avoids these pitfalls and can be easier to accept.

For clinicians in end-of-life care, personal support from family or friends can be grounding and affirming, but it can also lack the professional attunement needed to truly process trauma-laden experiences. Consistent with prior literature noting that chronic emotional engagement can lead to overidentification or psychological detachment (Sinclair et al. 2017), well-meaning conversations may inadvertently evoke overidentification with patients or their distress. This may lead to repeated mental replays of distressing events or blurring the boundaries between one’s professional role and personal life. This could explain why, in this sample, personal support was associated with emotional connection (and thus higher CS) but also with higher burnout, and why under high trauma exposure, it amplified rather than reduced STS. Additionally, some healthcare workers may occupy dual caretaking roles (e.g., providing emotional care to patients during their workdays and then assuming similar responsibilities at home for children, partners, or aging or ill family members). This parallel process can obscure the distinction between professional and personal caregiving, fostering overidentification with others’ suffering and limiting opportunities for emotional restoration. For these clinicians, the demands of constant empathy across settings may impact CS and heighten vulnerability to burnout and STS.

These findings also echo communal coping models, which show that when stress is shared within close personal networks, emotions can be contagious, especially when the stressor is chronic and high intensity. This does not mean personal support is harmful. It does mean that the form, responsiveness, and contextual fit of that support matter. For clinicians regularly encountering trauma, personal networks may need to be complemented by structured, peer-based, trauma-informed spaces that allow the content and emotional weight of clinical work to be processed without unintentionally increasing the burden.

### Demographic patterns and developmental considerations

Younger age and fewer years of experience were linked to greater burnout and STS, consistent with developmental models suggesting that early-career providers may have less established cognitive-emotional strategies for navigating sustained exposure (West et al. 2018). The lower burnout scores reported by Black participants warrant cautious interpretation, as they may reflect differences in coping strategies, sociocultural resilience factors, or potential underreporting due to stigma or occupational norms. Additionally, there was also a large discrepancy in the sample size between Black participants and White participants, which may have impacted this result.

Although research on the characteristics of those who choose to work in end-of-life care is limited, several studies suggest that these clinicians often hold strong values related to meaning-making, spirituality, and acceptance of mortality. Nurses and physicians in palliative and hospice care report higher intrinsic motivation toward helping roles and a belief in the importance of alleviating suffering, which may shape both their professional commitment and their emotional responses to patient loss (Peters et al. 2013; Sinclair et al. 2017). Some evidence from pediatric oncology further suggests that providers’ meaning systems and moral beliefs about death can influence how they process grief and sustain empathy (Boyle 2018). These findings underscore that clinicians drawn to end-of-life care may possess both heightened empathic sensitivity and a deep sense of purpose, factors that can serve as either protective or risk elements depending on systemic support and cumulative exposure. The absence of consistent differences across gender, education, professional role, or work setting suggests that vicarious trauma remains a broadly shared occupational hazard across demographic groups in these environments.

### Clinical and organizational implications

The findings carry several implications for sustaining empathic presence in high-trauma clinical environments. Individual-level interventions may include resilience-building programs, structured reflective practice, and evidence-based emotion regulation training could be integrated into professional development rather than offered as optional wellness add-ons (Duarte and Pinto-Gouveia 2017).

Organizational-level strategies such as institutional policies should prioritize visible, responsive leadership, meaningful recognition, and the consistent provision of resources that convey genuine valuing of staff contributions (Eisenberger et al. 1986; Killian 2008). Peer-based and trauma-informed support could also be highly beneficial given the nuanced risks associated with personal support (Bellehsen et al. 2024). Prior studies have shown that peer support can reduce emotional exhaustion, normalize trauma responses, and promote psychological safety among clinicians exposed to repeated patient suffering (Dekel and Nuttman-Shwartz 2009; Shapiro and Galowitz 2016; West et al. 2018). Organizations should therefore invest in formalized peer support programs, structured debriefings, and supervision models that allow safe, informed processing of traumatic events. Embedding these programs within existing institutional structures can also foster resilience and reinforce shared meaning that sustains empathic engagement in end-of-life care.

### Limitations and future directions

Several limitations warrant consideration. The cross-sectional design precludes causal inference, and self-report measures may be subject to recall and social desirability biases. The sample was drawn from a single health system in the northeastern United States, which may limit generalizability. Additionally, participation was voluntary, and the self-selecting nature of the sample may have attracted individuals with a stronger interest in or personal investment in topics related to well-being and trauma exposure. Findings may therefore overrepresent clinicians who are more reflective about these issues compared to the broader population of end-of-life care providers.

Future research should employ longitudinal designs to examine relationships among trauma exposure, protective factors, and ProQOL outcomes. Mixed-methods approaches could illuminate the specific mechanisms through which different forms of support influence well-being, particularly the conditions under which personal support functions as a buffer versus a risk factor. Intervention studies are also needed to test whether resilience training, organizational support enhancement, and trauma-informed peer programs produce sustained improvements in both CS and distress-related outcomes. For instance, the effectiveness of a structured peer support program with dedicated, protected time and a facilitator to openly discuss stressors may be a step toward supporting healthcare workers in end-of-life care.

## Conclusion

Vicarious trauma is a pervasive feature of end-of-life care and a potent predictor of burnout and STS. Resilience and organizational support appear to be broadly protective, while personal support demonstrates a more complex, and at times paradoxical, relationship with well-being. Furthermore, factors such as resilience and organizational support also contribute to sustaining CS, the sense of purpose and fulfillment that enable clinicians to remain meaningfully engaged despite repeated exposure to suffering. These findings suggest that sustaining empathy without compromising clinician health requires a multi-level approach that integrates individual skills development with systemic, trauma-informed organizational practices. Addressing these complexities proactively may help preserve both the human connection that underpins high-quality end-of-life care and the well-being of the clinicians who provide it.

## Data Availability

The data supporting the findings of this study are available from the corresponding author upon reasonable request. Data are not publicly available due to privacy or ethical restrictions.

## References

[ref1] Alkema K, Linton JM and Davies R (2008) A study of the relationship between self-care, compassion satisfaction, compassion fatigue, and burnout among hospice professionals. *Journal of Social Work in End-of-Life & Palliative Care* 4(2), 101–119. doi:10.1080/15524250802353934. 19042895

[ref2] Bellehsen MH, Cook HM, Shaam P, et al. (2024) Adapting the stress first aid model for frontline healthcare workers during COVID-19. *International Journal of Environmental Research & Public Health* 21(2), 171. doi:10.3390/ijerph21020171. 38397662 PMC10887691

[ref3] Boyle DA (2018) Reflections on the emotional hazards of pediatric oncology nursing: four decades of perspectives and potential. *Journal of Pediatric Nursing.* 40, 63–73. doi:10.1016/j.pedn.2018.03.007. 29776481

[ref4] Bride BE, Robinson MM, Yegidis B, et al. (2004) Development and validation of the Secondary Traumatic Stress Scale. *Research on Social Work Practice* 14(1), 27–35. doi:10.1177/1049731503254106.

[ref5] Cañadas-de la Fuente GA, Ortega E, Ramírez-Baena L, et al. (2018) Prevalence of burnout in oncology nursing: a systematic review and meta-analysis. *Psychooncology* 27(5), 1426–1433. doi:10.1002/pon.4632. 29314432

[ref6] Canty-Mitchell J and Zimet GD (2000) Psychometric properties of the Multidimensional Scale of Perceived Social Support in urban adolescents. *American Journal of Community Psychology* 28(3), 391–400. doi:10.1023/A:1005109522457. 10945123

[ref7] Dekel R and Nuttman-Shwartz O (2009) Secondary traumatization among social workers treating trauma survivors: the contributions of training, supervision, and workplace support. *British Journal of Social Work* 39(10), 233–247. doi:10.1093/bjsw/bcn104.

[ref8] Delaney MC (2018) Caring for the caregivers: evaluation of the effect of an eight-week pilot mindful self-compassion training program on nurses’ compassion fatigue and resilience. *PLoS One* 13(11), e0207261. doi:10.1371/journal.pone.0207261. 30462717 PMC6248952

[ref9] Duarte J and Pinto-Gouveia J (2017) Empathy and feelings of guilt and shame in nurses: a cross-sectional study of their role in burnout and compassion fatigue symptoms. *Applied Nursing Research* 35, 42–47. doi:10.1016/j.apnr.2017.02.006. 28532725

[ref10] Eisenberger R, Huntington R, Hutchison S, et al. (1986) Perceived organizational support. *Journal of Applied Psychology* 71(3), 500–507. doi:10.1037/0021-9010.71.3.500.

[ref11] Figley CR (1995) *Compassion Fatigue: Coping with Secondary Traumatic Stress Disorder in Those Who Treat the Traumatized*. Routledge.

[ref12] Granek L, Tozer R, Mazzotta P, et al. (2012) Oncologists’ experiences of grief following patient death: a qualitative study. *Psychooncology* 21(9), 1029–1037. doi:10.1002/pon.1993.

[ref13] Jenkins SR and Baird S (2002) Secondary traumatic stress and vicarious trauma: a validational study. *Journal of Traumatic Stress* 15(5), 423–432. doi:10.1023/A:1020193526843. 12392231

[ref14] Kadambi MA and Truscott D (2004) Vicarious trauma among therapists working with sexual violence, cancer, and general practice. *Canadian Journal of Counselling* 38(4), 260–276.

[ref15] Killian KD (2008) Helping till it hurts? A multimethod study of compassion fatigue, burnout, and self-care in clinicians working with trauma survivors. *Traumatology* 14(2), 32–44. doi:10.1177/1534765608319083.

[ref16] Lock S, Rees CS and Heritage B (2020) Development and initial validation of the State Assessment of Resilience Scale (STARS). *Australian Psychologist* 55(5), 497–510. doi:10.1111/ap.12456.

[ref17] Maisel NC and Gable SL (2009) The paradox of received social support: the importance of responsiveness. *Psychological Science* 20(8), 928–932. doi:10.1111/j.1467-9280.2009.02388.x. 19549083

[ref18] Orrù G, Marzetti F, Conversano C, et al. (2021) Secondary traumatic stress and burnout in healthcare workers during the COVID-19 outbreak. *International Journal of Environmental Research & Public Health* 18(1), 337. doi:10.3390/ijerph18010337. 33466346 PMC7794988

[ref19] Pereira SM, Fonseca AM and Carvalho AS (2011) Burnout in palliative care: a systematic review. *European Journal of Oncology Nursing* 15(5), 424–433. doi:10.1016/j.ejon.2010.11.003. 21558108

[ref20] Peters L, Cant R, Payne S, et al. (2013) How death anxiety impacts nurses’ caring for patients at the end of life: a review of literature. *The Open Nursing Journal* 7, 14–21. doi:10.2174/1874434601307010014. 23400515 PMC3565229

[ref21] Rhoades L and Eisenberger R (2002) Perceived organizational support: a review of the literature. *Journal of Applied Psychology* 87(4), 698–714. doi:10.1037/0021-9010.87.4.698. 12184574

[ref22] Shapiro J and Galowitz P (2016) Peer support for clinicians: a programmatic approach. *Academic Medicine* 91(9), 1200–1204. doi:10.1097/ACM.0000000000001297. 27355784

[ref23] Sinclair S, Raffin-Bouchal S, Venturato L, et al. (2017) Compassion fatigue: a meta-narrative review of the healthcare literature. *International Journal of Nursing Studies* 69, 9–24. doi:10.1016/j.ijnurstu.2017.01.003. 28119163

[ref24] Stamm BH (2010) *The Concise ProQOL Manual (Version 5)*. ProQOL.org. https://proqol.org/proqol-manual.

[ref25] Sutton L, Webster L and West M (2022) The contribution of organisational factors to vicarious trauma, secondary traumatic stress and compassion fatigue in mental health professionals: a systematic review. *European Journal of Psychotraumatology* 13(1), 2018794. doi:10.1080/20008198.2021.2018794. PMC8820814 35140879

[ref26] Vrklevski L and Franklin J (2008) Vicarious trauma: the impact on solicitors of exposure to traumatic material. *Traumatology* 14(1), 106–118. doi:10.1177/1534765607313044.

[ref27] West CP, Dyrbye LN and Shanafelt TD (2018) Physician burnout: contributors, consequences and solutions. *Journal of Internal Medicine* 283(6), 516–529. doi:10.1111/joim.12752. 29505159

[ref28] Zimet GD, Dahlem NW, Zimet SG, et al. (1988) The Multidimensional Scale of Perceived Social Support. *Journal of Personality Assessment* 52(1), 30–41. doi:10.1207/s15327752jpa5201_2.

